# Embolization of ruptured pancreaticoduodenal arcade aneurysms due to median arcuate ligament without celiac artery revascularization: a single-center experience and literature review

**DOI:** 10.1186/s42155-025-00534-1

**Published:** 2025-03-17

**Authors:** Rémi Grange, Nicolas Magand, Noémie Lutz, Bertrand Le Roy, Claire Boutet, Sylvain Grange

**Affiliations:** 1 Department of Radiology, University Hospital of Saint-Etienne, Avenue Albert Raimond, Saint-Priest-En-Jarez, 42270 France; 2 Department of Oncologic and Digestive Surgery, University Hospital of Saint-Etienne, Saint Priest-en-Jarez, 42270 France

**Keywords:** Aneurysm, Celiac artery, Bleeding, Embolization

## Abstract

**Background:**

The aim of this single-center retrospective study is to evaluate the feasibility, complications, and outcomes of transarterial embolization (TAE) for ruptured pancreaticoduodenal arcades aneurysms (PDAAs) due to median arcuate ligament (MAL), without subsequent revascularization of celiac artery (CA) occlusion/stenosis.

**Methods:**

Between January 1^st^ 2012 and June 1^st^ 2024, all records from adult patients (≥ 18 years old) referred to our hospital for TAE due to ruptured PDAAs were retrospectively reviewed. All referrals were based on emergency clinical decisions and computed tomography. Procedure data included procedure, type of embolic agent and per-procedural complication. TAE technical success was defined as the cessation of aneurysm opacification immediately after the TAE, based on angiographic findings. Overall technical success was defined as the cessation of aneurysm opacification after TAE or percutaneous salvage approach during the same session. Then, we analyzed all published original articles published between January 2007 and December 2024 on emergency TAE of ruptured PDAAs due to MAL, without subsequent treatment of CA stenosis/occlusion.

**Results:**

Nine patients (4 males) were referred for TAE for ruptured PDAAs due to MAL in our center. TAE technical success was achieved in 7/9 patients, and overall technical success was achieved in all patients. There were no major complications. No patients had rebleeding during follow-up.

We reviewed four retrospective studies including 29 patients treated for ruptured PDAAs due to MAL without subsequent treatment of CA stenosis/occlusion. No patient received additional treatment for CA stenosis/occlusion. No aneurysm recurrence was diagnosed during the reported follow-up periods ranging from 1 to 65 months.

**Conclusion:**

TAE for ruptured PDAAs without CA revascularization is safe and should be considered, although further studies are required to validate its validity and long-term outcomes.

## Background

The pancreaticoduodenal arcades (PDA) ensure a rich blood supply to the pancreas and duodenum, providing collateral circulation between the celiac artery (CA) and the superior mesenteric artery (SMA). The anterior arcade is formed by anastomoses between the anterior branches of the superior pancreaticoduodenal artery and the inferior pancreaticoduodenal artery, while the posterior arcade is formed by anastomoses between the posterior branches of these same arteries. Pancreaticoduodenal arcade aneurysms (PDAAs) are rare, accounting for only 0.2% of visceral aneurysms, but they can be fatal when ruptures occur [1, 2]. These aneurysms may arise secondary to atherosclerotic stenosis of the CA or as a result of median arcuate ligament (MAL). The MAL consists of a band of fibrous tissue connecting anteriorly the diaphragmatic crura surrounding the aortic hiatus [3]. CA stenosis/occlusion leads to the development of collateral vessels, causing hemodynamic alterations and increased flow in the PDAs, which may result in the formation of one or multiple PDAAs. It can also cause chronic abdominal pain, known as MAL syndrome [4, 5]. Contrary to pseudoaneurysms, true aneurysms result from a dilation of the vessel wall that involves all three layers of the vessel (the intima, media, and adventitia).In patients with uncomplicated PDAA and acceptable operative risk, treatment is recommended regardless of the aneurysm size due to the risk of rupture [6]. However, in the absence of pain suggestive of MALS, aneurysm rupture and haemorrhagic symptoms may be the first manifestations revealing the presence of PDAA. Traditionally, PDAAs have been treated surgically with aneurysm ligation. However, this approach is associated with significant morbidity. Transarterial embolization (TAE) has recently emerged as the first-line treatment for ruptured PDAAs [6–10]. However, treating multiple PDAAs can lead to parenchymal ischemia due to the retrograde blood flow through the PDAs [11, 12]. Furthermore, the risk of recurrence after TAE remains unknown.

Literature on the management of ruptured PDAAs due to MAL is limited. Existing studies include retrospective, heterogeneous series, including patients without CA stenosis, CA stenosis of atherosclerosis origin, and patients with MAL. These studies also include both treatments of unruptured and ruptured PDAAs [7–10, 13]. Due to the risk of recurrence, the Society of Vascular Surgery recommend to treat CA stenosis/occlusion after TAE of ruptured/unruptured PDAAs, even without MALS [6, 14]. However, the necessity of such aggressive strategies, especially even after treatment of ruptured PDAAs, is not known.

The aim of this single-center retrospective study is to evaluate the feasibility, complications, and outcomes of TAE for ruptured aneurysms of PDAs without subsequent treatment of CA occlusion/stenosis.

## Methods

### Study population

Between January 1, 2012 and June 1, 2024, all records from adult patients referred to our hospital for TAE for ruptured PDAAs due to MAL were retrospectively reviewed. Referrals were based on emergency clinical decisions and CT scans. The inclusion criterion was all adult patients (≥ 18 years old) referred for ruptured PDAAs due to CA stenosis/occlusion associated with MAL without subsequent treatment of CA stenosis/occlusion. The exclusion criteria were patients lost to follow-up and those with ruptured pseudoaneurysms from any cause. This study was approved by the Ethics committee of our institute (CHU de Saint-Etienne “Terre d’Ethique”, IRBN372025).

### Clinical data

We collected data from electronic medical records. Patient demographics included age, gender, and comorbid conditions before the TAE. Comorbid conditions included high blood pressure, chronic kidney disease, coronaropathy, anticoagulation or antiplatelet treatments. Biological data included TP, INR, platelet count, transfusion requirements and the quantity of transfused RBC units. Imaging data included computed tomography (CT) findings, angiographic findings, and CT imaging follow-up.

Procedure data included duration, type of embolic agent and per-procedural complication. Post procedure data included occurrence of minor and major complications, early and late rebleeding, type of management of rebleeding, length of hospitalization, and mortality.

### Pre-procedure

All patients underwent an abdominal CT scan (SOMATOM DEFINITION AS 64, Siemens AG, Medical Solution, Erlangen, Germany). Patients received ≥ 90 mL contrast medium (Xenetix 350, Guerbet, Villepinte, France) with a flow rate ≥ 3 ml/s. Unenhanced and contrast-enhanced abdominal CT at the arterial and portal phases were performed according to our hospital’s standard-of-care protocol. A haemorrhage was considered to be active when iodinated contrast leakage was present in the arterial phase and increased in the portal phase. The aneurysm was defined as an enlargement of all three layers of the artery, visible on the CT scan as a enlargement of the arterial calibre, stable at portal phase. Furthermore, the CT scan ruled out a cause that would suggest a pseudoaneurysm. PDAAs were defined as fusiform or sacciform.

### Transarterial TAE methods and techniques

**Fig. 1:**
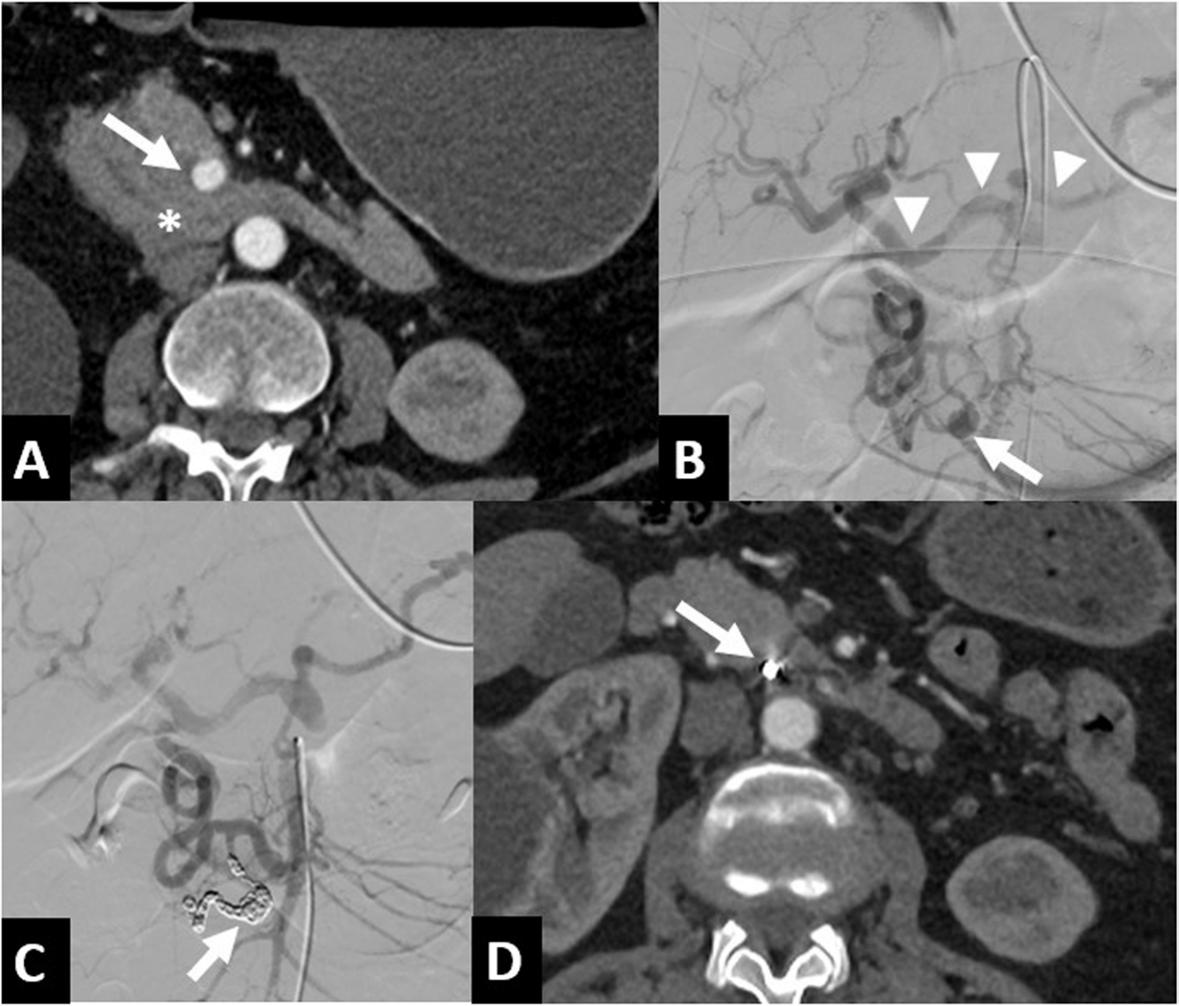
70 year-old female referred to emergency room for abdominal pain (Patient n°3). **A** The abdominal CT scan in axial section at arterial phase revealed a retro-portal hematoma (*) and a 12-mm saccular PIPDA aneurysm (white arrow). **B** Angiography of the SMA confirmed the aneurysm (white arrow) and demonstrated retrograde vascularization of CA branches (arrow head). **C** After occlusion and packing using 7 microcoils, the CT scan showed no complications (white arrow). **D** Four months later, the follow-up abdominal CT scan in axial section at arterial phase confirmed satisfactory occlusion, with no evidence of recurrence (white arrow). Abbreviations: CA, Celiac artery; CT, Computed tomography; PIPDA, Posteroinferior pancreaticoduodenal artery; SMA, Superior mesenteric artery

**Fig. 2:**
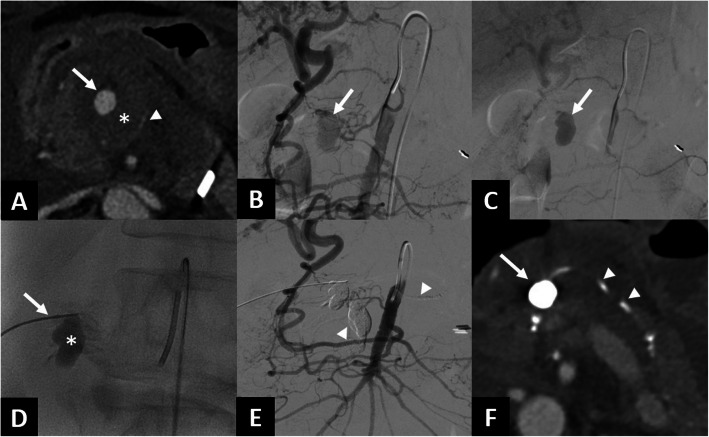
79-year-old male referred to the emergency room for abdominal pain (Patient n°4). **A** The abdominal CT scan in the axial section at arterial phase revealed a retro-pancreatic hematoma (*) resulting in compression of superior mesenteric vein (arrowhead) and a saccular aneurysm of the dorsal pancreatic artery (arrow). **B** Angiography of the SMA using a Sidewinder catheter confirmed the saccular aneurysm and demonstrated retrograde vascularization of the liver. **C** The aneurysm showed persistent late opacification (arrow). **D** After the technical failure of TAE, the aneurysmal sac (*) was punctured under ultrasound guidance using a 22G needle (arrow). **E** After percutaneous embolization using a Glubran®/Lipiodol® mixture, opacification of the SMA no longer showed opacification of the aneurysm and revealed non-target embolization (arrowheads). **F** The follow-up CT scan one week later confirmed satisfactory occlusion with Lipiodol® deposition, despite non-target embolization along the DPA. Abbreviations: CT, Computed tomography; DPA, Dorsalpancreatic artery; SMA, Superior mesenteric artery; TAE, Transarterial embolization

Procedures were performed under fluoroscopic guidance by one of four interventional radiologists whose experience ranged from 3 to 10 years. Patients were placed in a supine position. The right common femoral artery was accessed in all cases. Selective catheterism of CA and SMA was performed to determine active bleeding using a 4F Side catheter and a hydrophilic guidewire (Terumo®, Tokyo, Japan). Supraselective catheterism was performed, using a 2.7F microcatheter (Progreat®, Terumo, Tokyo, Japan). Occlusions of high-flow aneurysms were performed using N-butyl-2-cyanoacrylate (NBCA) (Glubran2®, GEM, Viareggio, Italy) or microcoils (Interlock®, Boston Scientific) (Fig. 1). In cases of embolization with NBCA, the microcatheter was flushed with Glucose 5%; the mixture of NBCA and ethiodized oil (Lipiodol®, Villepinte, Guerbet, France) was made using 10 ml luer lock syringes and a 3-way tap, and then loaded into a 3 mL syringe. In case of embolization with microcoils, sandwich occlusion was preferred in order to prevent recurrence via anastomosis. After TAE, complete angiograms were performed to confirm that bleeding had been successfully controlled (Fig. 2).

In case of technical TAE failure, echography was used to visualize the aneurysm. Aneurysms were then punctured under ultrasound guidance, using a 22G-needle. Under fluoroscopic guidance, iodine-contrast agent was injected, allowing for the confirmation of the correct needle position. After flushing the needle with Glucose 5%, a 0.5 mL mixture of Glubran2®/Lipiodol® was injected in a 1/1 ratio, until there was complete opacification of the aneurysm. The needle was removed after approximatively one minute. All patients were monitored in intensive care units after the procedure. Right femoral access was kept for 24 h. The procedure time was defined as the duration, in minutes, between the first and last scopy image.

### Patient follow-up

We evaluated data on patients’ long-term outcomes, including incidence of rebleeding, mortality, and procedure-related complications. Due to the technical complexity of embolizing high-flow aneurysms, a CT scan was systematically performed after the procedures. Suspicion of rebleeding was based on assessment of haemoglobin levels, and imaging showing recurrences of bleeding.

TAE technical success was defined as the cessation of aneurysm opacification immediately after the TAE, based on angiographic findings. Overall technical success was defined as the cessation of aneurysm opacification after TAE or percutaneous salvage approach during the same session. Clinical success was defined as resolution of signs and symptoms of bleeding during the 30-day follow-up period after TAE and no required surgery, nor repeat TAE.

Complications were defined as per operative complications if they occurred during the TAE and post-operative complications if they occurred during the follow-up period. Minor and major complications were separated using the CIRSE classification [15]. Grades 2, 3 were considered minor complications and grades 4, 5 and 6 were considered major complications. Grade 1 complication was not considered as a complication. Overall survival was defined as the number of days from the procedure to the date of latest available information or death.

### Statistical analysis

Results are presented as medians and inter-quartiles for continuous variables and as numbers and frequencies for categorical variables.

### Review of literature

We reviewed and analyzed all cases from original articles published on Pubmed between January 1, 2007 and December 1, 2024 related to emergency TAE of ruptured PDAAs due to MAL, without subsequent treatment of CA stenosis/occlusion. We excluded case reports and articles with missing data.

## Results

### Retrospective study

Between January 1, 2012 and January 1, 2024, 9 patients (4(44.4%) men/5(55.6% women) were referred for TAE for ruptured PDAAs due to MAL in our center, without subsequent treatment of CA occlusion/stenosis.

#### Patient characteristics

Patient characteristics are presented in Table 1. The median age was 74[67–81] years old. Initial symptoms were related to abdominal pain in 9/9 cases. Hemodynamic instability was recorded in 5/9(55.5%) patients. The median hemoglobin level was 9.8[9–12.2] g/dl. All patients had a pre-operative angiographic abdominal CT. Active bleeding was detected on CT scan in 5/9(55.5%) patients. Hemoperitoneum was localized in retroperitoneum in all cases. The median size of aneurysms was 10[8–12] mm. Seven out of nine (77.8%) aneurysms were fusiform and 2/9(22.2%) were sacciform.

**Table 1:** Population characteristics

Patient number	Patient age, sex	Hb nadir (g/dl)	RBC transfusion	Location of aneurysm	Size of aneurysm (mm)	Type of aneurysm
1	89, M	10.0	1	PIPDA	10	Fusiform
2	66, M	14.1	0	AIPDA	7	Fusiform
3	70, F	12.9	0	PIPDA	12	Sacciform
4	79, M	9.0	1	DPA	24	Sacciform
5	74, F	6.8	1	DPA	11	Fusiform
6	67, F	9.8	0	AIPDA	7	Fusiform
7	81, F	9.3	1	PIPDA	8	Fusiform
8	66, F	8.4	1	DPA	16	Fusiform
9	85, M	12.2	0	AIPDA	8	Fusiform

*Abbreviations: AIPDA* Anteroinferior pancreaticoduodenal artery, *CA* Celiac artery, *DPA* Dorsal pancreatic artery, *Hb* haemoglobin, *NBCA* N-Butyl-Cyanoacrylate, *PIPDA* Posteroinferior pancreaticoduodenal artery, *RBC* Red Blood Cell

#### Procedural data

Catheterism was performed by SMA in 7/9(77.8%) patients. TAE technical success was achieved in 7/9(77.8%) patients, and overall technical global success was achieved in 9/9 patients. In two patients, the feeding artery was not visible on angiogram, and they were treated by percutaneous approach using Glubran/Lipiodol mixture. Per-procedure data are presented in Table 2. The median procedure duration was 102.5[87.5–122.5] minutes, and 4/9(44.4%) patients were treated with microcoils alone.

**Table 2:** Procedural data of the study population (*n* = 9). Abbreviations: NBCA, N-Butyl-Cyanoacrylate

Patient number	Embolization	Procedure duration (minutes)	Approach	Embolic agent	TAE technical success	Overall technical success ^a^	Clinical success	Early complications	Clinical follow-up	CT follow-up (months)
1	Occlusion	80	SMA	NBCA	Yes	Yes	Yes	No	2	2
2	Occlusion + Sandwich	90	SMA	NBCA + Microcoils	Yes	Yes	Yes	No	16	2
3	Sandwich + Packing	80	SMA	Microcoils	Yes	Yes	Yes	No	4	4
4	Occlusion	120	SMA	NBCA	No → percutaneous salvage approach	Yes	Yes	Yes Grade 1 non-target embolization	5	4
5	Occlusion	90	SMA	NBCA	No → percutaneous salvage approach	Yes	Yes	No	4	4
6	Sandwich	130	SMA	Microcoils	Yes	Yes	Yes	No	15	9
7	Sandwich	160	SMA	Microcoils	Yes	Yes	Yes	No	85	85
8	Sandwich	115	SMA	Microcoils	Yes	Yes	Yes	No	53	18
9	Occlusion	80	CA	NBCA	Yes	Yes	Yes	Yes Grade 1 non-target embolization	50	37

*Abbreviations*: *CA* Celiac artery, *Hb* haemoglobin, *NBCA* N-Butyl Cyanoacrylate, *SMA* Superior mesenteric artery

^a^Defined as the cessation of aneurysm opacification after TAE or percutaneous salvage approach during the same session

#### Clinical outcomes

There were 2 grade 1 complications, due to non-target embolizations along DPA and right gastroepiploic artery (Patient numbers 4 and 9, respectively), without clinical consequences. There were no major complications. Clinical success was achieved in 9/9 patients. The median CT follow-up time was 4[2–18] months, and the median clinical follow-up time was 15[4–50] months. No patients had PDAA recurrence during CT follow-up or rebleeding during follow-up. There were no deaths 30 days post-procedure. One patient (Patient 1) died two months after TAE, which was related to sliding syndrome, and there were no signs of recurrent bleeding.

### Review of literature

We reviewed four retrospective studies covering 29 patients treated for ruptured PDAAs due to MAL without subsequent treatment of CA stenosis/occlusion [7–10]. There were 3 monocentric retrospective studies and one bi-centric retrospective study. Combining all studies, the median age was 64[56–73] years, including 12/29(41.3%) patients ≥ 70 year-old. The median aneurysm size was 8[5–12.75] mm, and 20/30(66.6%) aneurysms were < 10 mm. Coils alone were used in 21/29(72.4%) procedures, and the sandwich technique alone was used for 19/29(65.5%) patients. The technical success rate was 25/29(86.2%), and the clinical success rate was 27/29(93.1%). Suzuki et al. recorded 3 technical failures, which were defined by persistent opacification of the aneurysm at the end of procedure, but with complete thrombosis at 1 month CT follow-up. Four early complications were recorded, related to CA and iliac artery dissection (*n* = 2) and non-target coil and NBCA migration (*n* = 2). There was a major complication related to the non-target embolization of NBCA, which led to sepsis and required a splenectomy. No patient received additional treatment for CA stenosis/occlusion. No aneurysm recurrence was diagnosed after a follow-up period ranging from 1 to 65 months. Further information of patient characteristics and procedures is detailed in Table 3.

**Table 3:** Patient characteristic and procedural data of 29 patients in literature review treated for ruptured PDAAs due to MAL without subsequent treatment of CA occlusion/stenosis

Reference	Patient age, sex	MAL with CA stenosis/occlusion	Location of aneurysm	Presentation	Size of aneurysm (mm)	Embolization	Approach	Embolic agent	Technical success	Clinical success	Early complications	Length of follow-up (Months)	Recurrence
Suzuki et al. [10]	55, F	Yes	APDA	Rupture	9	Sandwich	SMA / CA	Microcoils	No^a^	Yes	No	5–65 (Range)	No
	60, F	Yes	DPA	Rupture	5	Sandwich + Packing	SMA	Microcoils	Yes	Yes	No		No
	79, M	Yes	DPA	Rupture	8	Sandwich	SMA	Microcoils	No^a^	Yes	No		No
	43, M	Yes	DPA	Rupture	5	Aff	SMA / CA	Microcoils / NBCA	No^a^	Yes	No		No
	56, M	Yes	PPDA	Rupture	3	Sandwich + Packing	SMA	Microcoils	Yes	Yes	No		No
	73, F	Yes	PPDA APDA	Rupture	7 6	Sandwich + Packing	SMA / CA	Microcoils / Gelatine sponge	Yes	Yes	No		No
Siauve et al. [7]	56, M	Yes	PIPDA	Rupture	13	Packing	SMA	Onyx®	Yes	Yes	No	1	No
	73, M	Yes	PIPDA	Rupture	8	Packing	SMA	NBCA	Yes	No	No	/	No
	70, M	Yes	AIPDA	Rupture	15	Packing + Sandwich	SMA	Microcoils + Onyx®	Yes	Yes	Right iliac artery dissection	18	No
	82, M	Yes	APDA	Rupture	9	Sandwich	SMA	Microcoils	No	No	CA ostium dissection	/	No
	66, F	Yes	AIPDA	Rupture	8	Sandwich	SMA	Onyx®	Yes	Yes	No	< 1 month	No
Jazzar et al. [8]	87, F	Yes	NA	Rupture	16	Sandwich	SMA	Microcoils	Yes	Yes	No	14	No
	64, M	Yes	NA	Rupture	4	Sandwich	SMA	Microcoils	Yes	Yes	No	51	No
	58, F	Yes	NA	Rupture	12	Sandwich	SMA	Microcoils	Yes	Yes	No	37	No
	53, F	Yes	NA	Rupture	15	Sandwich	SMA	Microcoils	Yes	Yes	No	16	No
	63, M	Yes	NA	Rupture	15	Packing	SMA	Microcoils	Yes	Yes	No	65	No
	64, M	Yes	NA	Rupture	26	Occlusion	SMA	NBCA	Yes	Yes	Glue migration → Sepsis → Splenectomy	1	No
	47, F	Yes	NA	Rupture	34	Sandwich	SMA	Microcoils	Yes	Yes	No	2	No
	80, F	Yes	NA	Rupture	5	Occlusion	SMA	Onyx®	Yes	Yes	No	27	No
	83, M	Yes	NA	Rupture	9	Sandwich	SMA	Microcoils	Yes	Yes	Coil migration in HA	3	No
	77, M	Yes	NA	Rupture	18	Sandwich	SMA	Microcoils	Yes	Yes	No	1	No
Chivot et al. [9]	72, M	Yes	DPA	Rupture	2	Sandwich	CA	Microcoils	Yes	Yes	No	48	No
	52, M	Yes	PPDA	Rupture	4	Sandwich	CA	Microcoils	Yes	Yes	No	36	No
	71, F	Yes	PPDA	Rupture	3.5	Sandwich	SMA	Microcoils	Yes	Yes	No	27	No
	61, F	Yes	APDA	Rupture	6.5	Sandwich	SMA	Microcoils	Yes	Yes	No	11	No
	61, M	Yes	PPDA	Rupture	5	Sandwich	SMA	Microcoils	Yes	Yes	No	10	No
	42, M	Yes	DPA	Rupture	6	Sandwich	SMA	Microcoils	Yes	Yes	No	8	No
	38, F	Yes	DPA	Rupture	10	Sandwich	SMA	Microcoils	Yes	Yes	No	5	No
	78, F	Yes	DPA	Rupture	4	Sandwich	CA	Microcoils	Yes	Yes	No	5	No

*Abbreviations*: *Aff* Afferent, *AIPDA* Anteroinferior pancreaticoduodenal artery, *APDA* Anterior pancreaticoduodenal artery, *CA* Celiac artery, *DPA* Dorsal pancreatic artery, *HA* hepatic artery, *PIPDA* Posteroinferior pancreaticoduodenal artery, *PPDA* Posterior pancreaticoduodenal artery, *MAL* median arcuate ligament, *NA* not available, *NBCA* N-Butyl Cyanoacrylate, *SMA* Superior mesenteric artery

^a^In Suzuki et al.’s study, technical success was defined by the persistent opacification of the aneurysm at the end of the procedure

## Discussion

In line with the literature review presented, the present study demonstrates that emergency TAE of PDAAs is associated with a high rate of overall technical and clinical success. Even without subsequent treatment of CA occlusion/stenosis, no recurrent PDAAs were recorded on follow-up CT imaging, even after long-term follow-up.

A recent study conducted by Jazzar et al. showed that TAE of high-flow PDAAs was associated with no recurrence, even for the 10 patients treated for ruptured PDAAs [8]. The authors concluded that TAE alone, without revascularization CA stenosis/occlusion, is sufficient. Similarly, Siauve et al. reported two deaths out of five patients treated for ruptured PDAAs, but none of the surviving patients showed signs of recurrence during follow-up [7]. To our knowledge, no bleeding recurrence after TAE of ruptured PDAAs has been documented. Nevertheless, Tamura et al. reported that 1/23(4.3%) patients treated for unruptured PDAAs developed de novo PDAA one year after treatment of inferior pancreaticoduodenal artery, and was managed conservatively. One/23(4.3%) patient developed rapid enlargement of untreated PDAA 3 months after endovascular trapping and was treated by embolization [16]. This phenomenon may occur due to flow redirection caused by the occlusion of a pancreaticoduodenal branch, potentially leading to the formation of new PDAAs. Despite these 2 recurrence/rapid enlargement PDAA after TAE, no haemorrhage was observed. CT imaging is therefore necessary to follow these patients after emergency TAE. The decision to treat any PDAA recurrence should consider the benefit-risk balance, particularly the risk of parenchymal ischemia following repeat TAE. Moreover, to our knowledge, no study has determined whether patients treated for a ruptured PDAA due to MAL have an increased risk of haemorrhage from PDAA recurrence. In Tamura et al.’s study, 4/23(17.4%) patients benefited from adjunctive angioplasty for CA occlusion, including one case of stent occlusion occurring one month after the procedure, without clinical worsening [16].

The subsequent management of CA stenosis/occlusion secondary to MAL after TAE of ruptured PDAAs remains a topic of debate [8, 17, 18]. Proposed approaches include laparoscopic techniques (e.g., MAL release or bypass), endovascular approaches (e.g. stenting) or a combination of laparoscopic surgery and endovascular interventions, which is referred to as a *hybrid technique* [18–23]. The potential benefit of this strategy is to prevent the risk of PDAA recurrence and potentially fatal haemorrhage in patients who have already experienced a bleeding episode. Schneider et al. treated 6 patients using a combined laparoscopic and endovascular approach following embolization for ruptured PDAAs caused by CA stenosis due to MAL [20]. In this series, two patients required retreatment for stent occlusion and restenosis, and the median hospital stay was 16 days. A laparoscopic approach requires general anesthesia, posing risks of vascular injury, open conversion, and significant durations of hospitalization. CA stenting alone is associated with a significant risk of technical failure, restenosis, and thrombosis, limiting its indication [17, 23, 24]. These poor outcomes may be due to the extrinsic compression of CA causing intimal hyperplasia resulting luminal narrowing of artery. CA stenting may be useful in adjunction with laparoscopic release in case of stenosis after operative intervention [3]. Combined approaches are frequently needed to achieve correct patency [22]. There are no robust data on PDAA recurrence after TAE, including after TAE of ruptured PDAAs. Moreover, the performance of abdominal CT in detecting PDAAs allows for the identification of even subcentimetric aneurysms. On the 29 patient cases described in our literature review, 12(41.4%) were ≥ 70 years old. In this population, risk of recurrence is lower, and operative risk is significant. Close monitoring by abdominal CT is likely preferred, particularly for older and frail patients.

Coils alone were the embolic agent of choice in our study and in the literature (21/29(72.4%)). It is recommended to treat patients who can be treated with coils as the first option [6]. They are easy to use, and the risk of non-target embolization is lower than with liquid agents, particularly in high-flow arteries. However, the use of NBCA has been particularly helpful in treating patients whose artery supplying the aneurysm is difficult to catheterize. The present study reveals some TAE technical failure. This aligns with the findings of Siauve et al., where no feeding arteries to the PDAA were identified in one patient, resulting in technical failure [7]. In such cases, a percutaneous approach plays a vital role, particularly in emergencies. It has already demonstrated its efficacy in treating intramuscular, pulmonary, or mesenteric pseudoaneurysms [25–27]. This approach is quick, cost-effective, and associated with a high rate of technical success [26, 28, 29]. To minimize the risk of non-target embolization, a high NBCA/Lipiodol emulsion ratio should be used. However, when non-target embolization does occur, the clinical consequences are usually limited. This percutaneous approach emphasizes the importance of multimodal strategies for treatment of ruptured aneurysm, using CT scan/scopic guidance, echography/scopic guidance. Thin 22G needles are sufficient for injecting the Glubran/Lipiodol mixture and reduce the risk of visceral injury in cases of transgastric or transduodenal punctures. Microcoils were used in the majority of the cases we reported. However, NBCA can play an important role when the arcades are particularly tortuous and the microcatheter is positioned at a distance from the DPAA. Its use should be based on the risk–benefit ratio. Jazzar et al. reported a case of splenectomy following unintended NBCA embolization [8].

The present study has some limitations. The present analysis included a small sample size, reflecting the rarity of this clinical presentation. Additionally, the study includes both long- and mid-term follow-up. Moreover, this approach has not been compared to cases with subsequent treatment of CA stenosis/occlusion after TAE of ruptured PDAAs.

## Conclusion

The present study demonstrates that TAE of ruptured PDAAs without subsequent treatment of CA stenosis/occlusion is both safe and effective. This approach should be considered, particularly in older patients. However, further studies are required to validate its effiacy and long-term outcomes.

## Data Availability

Data will be made available on reasonable request.

## Abbreviations

AIPDA: Anteroinferior pancreaticoduodenal artery
APDA: Anteroposterior pancreaticoduodenal artery
CA: Celiac artery
CT: Computed Tomography
DPA: Dorsal pancreatic artery
Hb: Haemoglobin
MAL: Median arcuate ligament
NA: Not available
NBCA: N-Butyl Cyanoacrylate
PDAA: Pancreaticoduodenal arcade aneurysm
SMA: Superior mesenteric artery
RBC: Red Blood Cell
